# Progress and challenges in the elimination of hepatitis C among people who inject drugs in Germany: results of a pilot study for a national monitoring system, 10 years after the first data collection

**DOI:** 10.1186/s12954-024-01119-2

**Published:** 2024-12-20

**Authors:** Gyde Steffen, Amrei Krings, Sarah Guttmann, Nadine Lübke, Kristin Meyer-Schlinkmann, Carsten Tiemann, Jörg Timm, Andreas Walker, Ruth Zimmermann, The DRUCK 2.0-study group, Markus Backmund, Hans-Peter Dorsch, Sebastian Bayer, Jörg Ciomber, Miriam Gerlich, Astrid Leicht, Martin Kießling, Esther Neumeier, Stine Nielsen, Willehad Rensmann, Dirk Schäffer, Olaf Ostermann, Christiane Stöter, Stefan Wiedemann, Katrin Wimmer

**Affiliations:** 1 Department of Infectious Disease Epidemiology, Robert Koch Institute, Berlin, Germany; 2 MVZ Labor Krone eGbR, Bad Salzuflen, Germany; 3 Institute of Virology, Heinrich-Heine-University, University Hospital, Düsseldorf, Germany; 4 Praxiszentrum Im Tal, Munich, Germany; 5 Aidsberatungsstelle Oberpfalz, Regensburg, Germany; 6 Fixpunkt e.V., Berlin, Germany; 7 Bundeszentrale Für Gesundheitliche Aufklärung, Cologne, Germany; 8 Mudra e.V., Nuremberg, Germany; 9 Deutsche Beobachtungsstelle für Drogen und Drogensucht, Munich, Germany; 10 Statens Serum Institut, Copenhagen, Denmark; 11 Aidshilfe Dortmund e.V., Dortmund, Germany; 12 Deutsche Aidshilfe e.V., Berlin, Germany; 13 Condrobs e.V., Munich, Germany; 14 Gemeinschaftspraxis Schlesisches Tor, Berlin, Germany; 15 Vista gGmbH, Berlin, Germany; 16 Drogenhilfe Schwaben gGmbH, Augsburg, Germany

**Keywords:** People who inject drugs, Prevalence, Hepatitis B, Hepatitis C, Infectious diseases, Germany

## Abstract

**Background:**

People who inject drugs (PWID) are at high risk of blood-borne infections, and injection drug use contributes significantly to hepatitis C virus (HCV) transmission. The WHO has therefore set targets of reducing HCV incidence and prevalence among PWID and increasing treatment coverage to eliminate HCV by 2030. The DRUCK study (2011–2014) found high HCV prevalence and low treatment coverage among PWID in Germany. To assess progress in the elimination of HCV among PWID, we conducted a cross-sectional study in two German federal states that piloted a future monitoring.

**Methods:**

PWID aged 16 + who injected drugs (previous 12 months) were recruited in low-threshold drug services and opioid agonist treatment (OAT) practices in Berlin and Bavaria between June 2021 and April 2022. Participants completed a questionnaire on sociodemographics, behaviours and access to care, and were tested for hepatitis B virus (HBV) and HCV, and HIV. Data was analysed regarding HCV prevalence, history of treatment, and risk and prevention behaviours. Results were compared with the DRUCK study.

**Results:**

A total of 588 PWID, with a median age of 39 (range: 17–66) years and 68% (399/587) male, were included in the analysis. Of the participants, 61% (353/574) reported receiving OAT and 14% (66/469) recent use of shared needles/syringes during the last 30 days. History of imprisonment was reported by 77% (444/577) and history of homelessness by 75% (428/569) of participants. Among anti-HCV positive participants, viraemic HCV infections decreased by 44% from 66% (904/1361) in 2011–2014 to 37% (160/432) in 2021–2022, while those with cleared HCV infection and treatment history increased from 20% (266/1361) to 34% (148/432).

**Conclusions:**

Despite a decrease since 2011–2014, viraemic HCV prevalence among PWID in Germany remains high, and treatment coverage is still insufficient. To achieve the WHO targets, universal health coverage and targeted integrated testing and treatment for PWID are needed. PWID receiving OAT and people in prison should be offered testing and treatment at any contact with the medical system. A nationwide monitoring system will help assess successes and remaining gaps, and track progress towards elimination of HCV among PWID in Germany.

**Supplementary Information:**

The online version contains supplementary material available at 10.1186/s12954-024-01119-2.

## Introduction

As part of the 2030 Agenda for Sustainable Development, the World Health Organization (WHO) called for elimination of sexually transmitted and blood-borne infections as a public health threat by 2030 [1, 2]. Targets were defined to reduce the incidence and mortality of hepatitis B and C virus (HBV, HCV), and human immunodeficiency virus (HIV) by diagnosing 90% of those infected and treating 80% of those diagnosed with HBV/HCV and 95% of those diagnosed with HIV [1, 3]. People who inject drugs (PWID) are particularly vulnerable to blood borne infections and injection drug use (IDU) contributes substantially to HCV transmission [4, 5]. The specific target of reaching a low HCV incidence of ≤ 2 / 100 person-years or an 80% reduction in viraemic HCV prevalence among PWID was set by WHO and the European Drugs Agency (EUDA) [6, 7]. Globally, there is an estimate of around 11 million PWID and 6.1 million of them are living with a viraemic HCV infection [8]. Important risk factors for HCV transmission are sharing of contaminated needles, syringes, or other drug paraphernalia, and history of imprisonment and homelessness, which are situations considered to promote higher risk behaviour and limit access to prevention [9–11]. In turn, high coverage of harm reduction measures including needle and syringe programmes (NSP) and opioid agonist therapy (OAT) prevent transmission by reducing sharing of needles and syringes as well as injecting frequencies [12, 13]. Early diagnosis and treatment of infected persons is crucial to avoid progressive liver disease and also plays a role in the approach of treatment as prevention [14–16]. However, due to structural and individual barriers, access to both is often limited for PWID [17–19]. As one of the priority groups for whom access to prevention and treatment should be ensured within the framework of national elimination efforts [3], PWID should be reached with a comprehensive package of the above-mentioned services [20, 21].

The German government has committed to the WHO elimination call with a national strategy, emphasizing the expansion of needs-oriented prevention and health services for PWID [22].

In Germany, a country with concentrated epidemics of sexually and blood-borne infections among at-risk groups, nearly half of new HCV infections are attributed to IDU [23] and overall, more than 130,000 people are estimated to inject drugs [24].

Between 2011 and 2014 a cross-sectional study was conducted in eight German cities (study acronym: DRUCK study) that included 2,077 PWID who had injected drugs within the last 12 months. These were recruited by respondent driven sampling (RDS) and examined in low-threshold drug services [25]. In addition to a detailed questionnaire-based interview, dried blood spots (DBS) from capillary blood were tested for HBV, HCV and HIV. The study revealed a high prevalence of viraemic HCV infections (44% HCV-RNA) [26], however the proportion of participants who reported history of HCV treatment was found to be low (25%).

In light of the alarming results of the DRUCK study, many low-threshold drug services in Germany intensified their prevention programs, e.g. regarding counselling, NSP and point of care testing (POCT) [27, 28]. However, there is a lack of recent and continuous data on the current HCV epidemic among PWID in Germany. Since the governance for health in Germany lies in the responsibility of the federal states or the communities there is no central programme nor the possibility to retrieve national data from one source.

In order to measure the achievement of the elimination targets in a standardized way, the WHO developed a monitoring and evaluation framework [29], which was further specified for PWID by the EUDA [7]. This resulted in a set of core and additional indicators (including HCV prevalence, change in viraemic HCV prevalence, needle/ syringe provision, OAT coverage, proportion of tested, treated and cured HCV infections, as well as HCV incidence) that are needed to encompass the epidemiological context, prevention needs, the continuum of care, and the impact of implemented services.

To assess elimination efforts’ progress of blood-borne and sexually transmitted infections among PWID in Germany, we developed a cross-sectional study design for a future monitoring system among PWID in sentinel cities, which was piloted in two federal states in 2021–2022 (study acronym DRUCK 2.0 study) [30]. With this analysis, based on data of the pilot study, we aim to assess the prevalence of viraemic and cleared HCV infection among PWID as well as reported treatment experience. Furthermore, the above-mentioned EUDA core indicators were used to assess prevalence of risk and preventive behaviour related to HCV. Prevalence was stratified by sociodemographic and behavioural factors to identify subgroups most at risk. Potential changes in HCV prevalence, treatment coverage and related behaviours were analysed by comparing the results obtained in 2021–2022 to the results from the DRUCK study conducted in 2011–2014.

## Methods

### Study design and population

Between June 2021 and April 2022 low-threshold drug services, including contact cafés, outreach services, drug consumption rooms (only available in Berlin) and drug counselling centres, and medical practices with OAT-services in the pilot federal states Berlin and Bavaria were invited to implement the DRUCK 2.0 study data collection in their routine work. We trained staff of these recruiting facilities that accepted our invitation (hereafter called facilities) to conduct all steps of the data collection and provided them with promotional material for recruitment. For the detailed recruitment strategy, see Krings et al. [30]. Inclusion criteria for study participants were age over 16 years, IDU during the last 12 months, and willingness to answer a questionnaire and give blood. Eligible clients of the facilities were informed about the study and undersigned a written consent.

The questionnaire was filled by all participants, containing 39 questions on sociodemographic characteristics, risk and protective behaviour and access to testing and care for sexually transmitted and blood-borne infections. The questionnaire was developed to cover the indicators recommended by WHO and EUDA [29, 31], and can be found here (www.rki.de/druck-studie).

Participants filled the questionnaire themselves or, depending on their own decision, with assistance of the facilities´staff. All study documents were written in plain language and translated to twelve different languages, based on the facilities’ needs. Language mediation via phone was available. Questionnaires were sent directly to the Robert Koch Institute (RKI).

After receiving test counselling, a capillary blood sample was collected as DBS from each participant on site and sent to a laboratory. Alternatively, in OAT practices venous blood samples were taken by the medical staff.

### Laboratory testing

At the laboratory, all samples were tested for both, Anti-HCV and HCV-RNA. Different DBS cards were used for serological (Ahlstrom-Munksjö no. 460) and PCR (Roche Diagnostics cobas® plasma separation cards) analysis. DBS cards passed a visual quality control to check proper soaking of the blood drops. For serological analysis, two spots à 6mm were punched per well and eluted by adding 225 µl Diluent (Roche Diagnostics) following incubation for 1 h at 400 rpm. After short centrifugation (5’, 4,000xg), analysis of anti-HCV was performed using Elecsys Anti-HCV II (Roche Diagnostics) on Cobas e 801 (Roche Diagnostics). For PCR analysis, deviating from the manufacturer's instructions, participants dripped blood from their fingertip directly onto the card (three spots per participant) instead of using a glass capillary. The dried plasma of two blood spots was extracted in 1700 µl SPER buffer (Specimen Pre-Extraction Reagent, Roche Diagnostics) followed by an incubation for 10 min at 56 °C and 1000 rpm. 1300 µl of the extract were used for analysis of HCV nucleic acids using the cobas®MPX (Multiplex HIV, HCV & HBV nucleic acid test, Roche Diagnostics) assay on the cobas® 6800 system. Serological analysis of venous blood samples from OST practices were carried out using the same test as for DBS samples, HCV viral load testing was performed using the Aptima™ HCV Quant Dx Assay (Hologic) on the Panther system.

Samples with detectable HCV RNA were sent to the National Reference Centre for HCV for genotyping and phylogenetic analysis. DBS were processed and genotyped as previously described [32]. In brief, 1–3 spots were punched out with a disposable punch, dissolved in ASL buffer and nucleic acid was extracted using the EZ1 Virus Mini Kit v2.0 on an EZ1 Advanced robot (Qiagen). After extraction, the core [33], env [34] and NS5A [35] regions were amplified and sequenced using Sanger sequencing as described.

Different options to inform study participants about their test results were offered. This is further explained in Krings et al. [30].

### Data collection

Data was collected pseudonymised using a unique identifier (ID) for all study documents and the blood samples. All participants received a 10 Euro voucher directly after participation.

At RKI, the questionnaires were entered electronically using Epidata (EpiData Association, Denmark), matched with the laboratory data via the ID and then anonymized. For all questions in the questionnaire, the responses "I don't know" and "I don't want to answer" were recoded as missed responses.

### Variables of interest

Viraemic HCV infection was defined as presence of HCV RNA (limit of detection approximately 1000 IU/ml for DBS, restricted by the quality of DBS, and 4 IU/ml for venous blood samples), regardless of anti-HCV presence, and cleared HCV infection as presence of anti-HCV in absence of HCV RNA (independent from reasons for clearance). History of HCV treatment was defined as reporting ever having received medication for HCV infection and, for validity reasons, testing positive for anti-HCV and/or HCV RNA.

### Statistical analysis

Analyses were performed using STATA 17 (StataCorp LLC, USA). Participants who did not meet the inclusion criteria or with missing HCV infection status (defined as either anti-HCV or HCV RNA or both missing) were excluded. Descriptive statistics were used to investigate HCV infection and treatment prevalence as well as the prevalence of risk and protective behaviour based on laboratory and questionnaire results. Descriptive analysis of continuous questionnaire variables was conducted calculating the median with minimum and maximum or alternatively creating categories. For categorial variables, categories were combined, if applicable. For stratified analyses proportions were displayed with 95% confidence intervals. Associations were considered significant if the 95% confidence intervals of the categories of interest did not overlap.

**Fig. 1:**
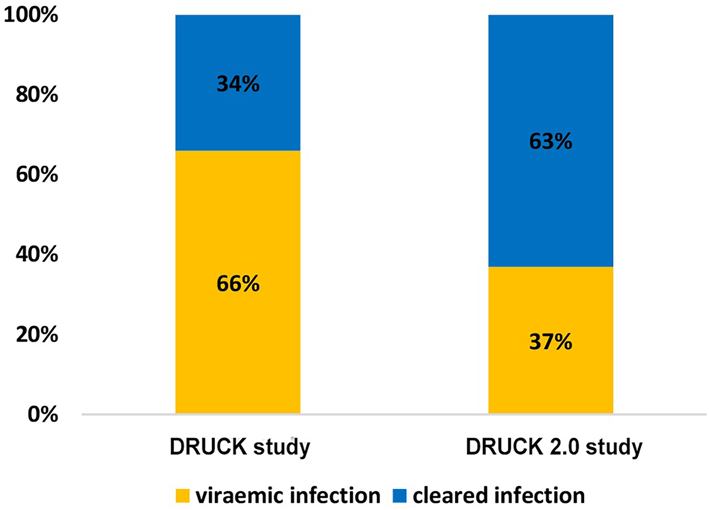
Proportion of participants with viraemic and cleared hepatitis C infection among anti-HCV positive participants, DRUCK study (2011–2014, N = 1.361) and DRUCK 2.0 study (2021–2022, N = 432)

**Fig. 2:**
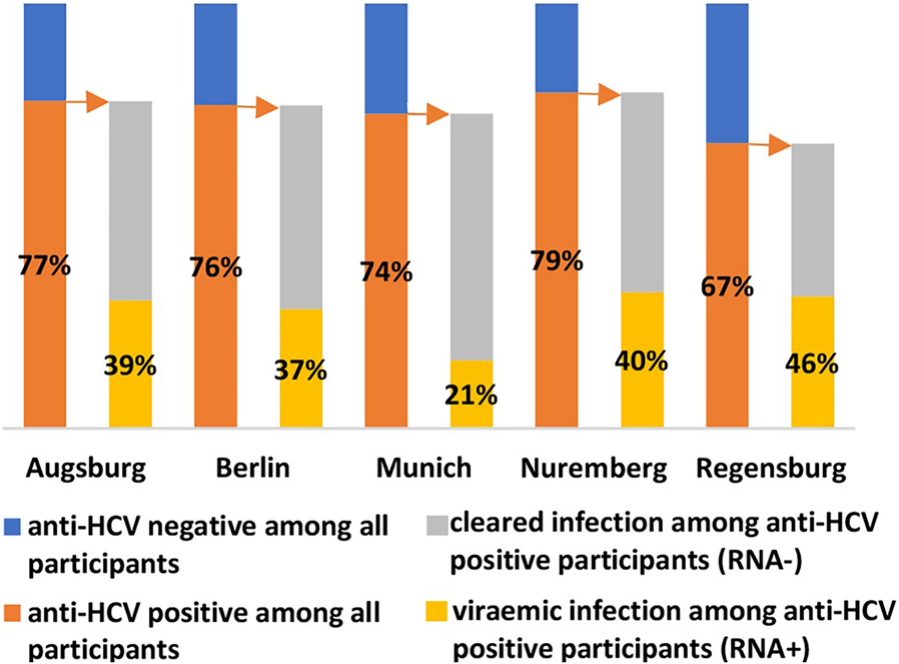
Proportion of anti-HCV positive participants among all participants (orange/ blue bars) and proportion of participants with viraemic hepatitis C infection among anti-HCV positive participants (yellow/ grey bars) by city of recruitment, DRUCK 2.0 study (2021–2022, number of participants: Augsburg N = 81, Berlin N = 143, Munich N = 105, Nuremberg N = 78, Regensburg N = 174)

**Fig. 3:**
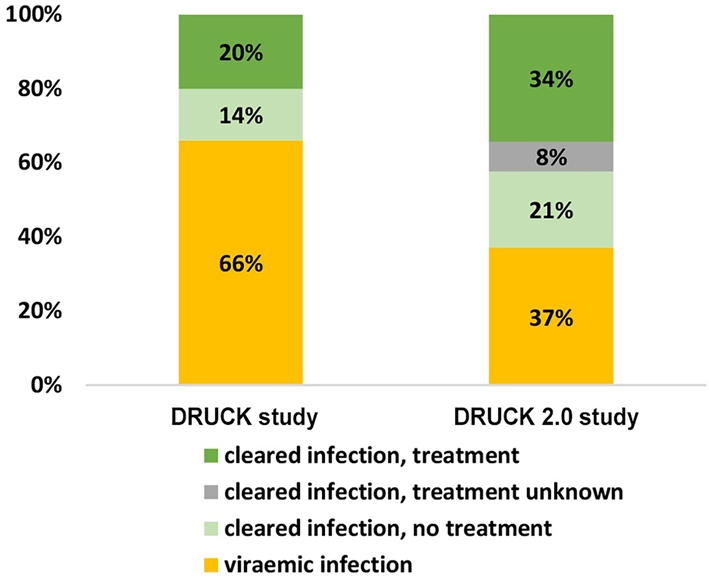
Self-reported HCV history of treatment (ever been treated) among anti-HCV positive tested participants, DRUCK-study (2011–2014, N = 1.361) and DRUCK 2.0 study (2021–2022, N = 432)

Prevalences of anti-HCV and viraemic HCV infection among all participants were separately stratified by sociodemographic and behavioural factors (Table 2). Proportions of anti-HCV positive participants among all participants and viraemic infections among anti-HCV positive participants were separately stratified by city of recruitment (Fig. 2). HCV infection status and history of treatment among anti-HCV positive participants are displayed (Figs. 1, 3). For comparison, results of the DRUCK study from 2011–2014 were used. The methodology and results of the DRUCK study have been described elsewhere [25]. Due to the different samples used for these two studies no test for association was used. The decrease in viraemic prevalence in the DRUCK 2.0 study population among anti HCV positives and among all participants compared to the respective categories in the DRUCK study (2011–2014) was calculated.

Ethical approval for the DRUCK 2.0 study was provided by the Medical Chamber Berlin (ETH-51/10).

## Results

Between June 2021 and April 2022 668 PWID were recruited in Berlin (n = 155) and Bavaria (n = 513). Recruitment took place in 20 drug services and three OAT practices. Overall, 596 participants (89%) fulfilled the inclusion criteria (81 in Augsburg, 146 in Berlin, four in Ingolstadt, 108 in Munich, 79 in Nuremberg, 175 in Regensburg, and three in Wurzburg). For 588 (99%) of them results for anti-HCV and HCV-RNA were available and the HCV infection status was determined. These were included in the analysis (see flowchart, additional file 1).

Of all included participants, 69% were recruited in contact cafés/outreach services, 19% in drug consumption rooms, 8.7% in OAT practices, and 3.6% in drug counselling centres. Median age of participants was 39 years (range: 17–66; n = 588), 68% were male and 22% were born abroad.

For detailed characteristics of the study participants, see Table 1.

**Table 1:** Recruitment details, sociodemographic and behavioural characteristics, hepatitis B-, hepatitis C- and HIV-status of the study population, DRUCK 2.0 study (2021–2022, N = 588)

N = 588		
Variable	n	%
**Blood sample method**		
Capillary blood/ dried blood spots	549	93
Venous blood/ serum	39	6.6
**City of recruitment**		
Augsburg	81	14
Berlin	143	24
Ingolstadt	4	0.5
Munich	105	18
Nuremberg	78	13
Regensburg	174	30
Wurzburg	3	0.5
**Setting of recruitment**		
Contact cafés/ outreach services	404	69
Drug consumption rooms	112	19
Drug counselling centres	21	3.6
OAT practices	51	8.7
Age group*		
< 25 years	31	5
25–39 years	278	47
40 years and older	279	47
Gender*		
Men	399	68
Women	184	31
Diverse	4	0.7
Country of birth*		
Germany	458	78
Abroad	130	22
UN-birth region, if born abroad*		
Northern Europe	4	3.2
Western Europe	6	4.8
Eastern Europe	52	42
Southern Europe	17	14
Outside Europe	45	36
Years with residence in Germany, if born abroad*		
Less than 5 years	7	5.6
5–9 years	20	16
10 years or more	97	78
History of homelessness*		
Yes	428	75
No	141	25
*Currently*	*139*	*25*
*Not currently/ never*	*408*	*75*
History of imprisonment*		
Yes	444	77
No	133	23
*Within last 12 months*	*146*	*25*
*Not within last 12 months/ never*	*427*	*75*
Injection drug use during last imprisonment, if history of imprisonment*		
Yes	105	24
No	329	76
Injection drug use within the last 30 days*		
Yes	487	84
No	93	16
Drug used most often within the last 30 days: Opioid*		
Yes	376	70
No	160	30
Drug used most often within the last 30 days: Cocaine*		
Yes	76	14
No	459	86
Drug used most often within the last 30 days: Methamphetamine*		
Yes	44	8.2
No	491	92
History of overdose with unconsciousness*		
Yes	390	69
No	177	31
*Within last 30 days *^*§*^	*48*	*10*
*Not within last 30 days/ never *^*§*^	*421*	*90*
Years since first drug injection*		
Less than 2 years	32	5.6
2–4 years	42	7.4
5 years or more	497	87
History of shared needles/ syringes*		
Yes	320	57
No	242	43
*Within last 30 days *^*§*^	*66*	*14*
*Not within last 30 days/ never *^*§*^	*403*	*86*
History of shared spoons, filters, water for drug preparation*		
Yes	405	72
No	161	28
*Within last 30 days *^*§*^	*138*	*29*
*Not within last 30 days/ never *^*§*^	*335*	*71*
Use of sterile needle for the last injection*^§^		
Yes	444	93
No	35	7
History of opioid agonist therapy (OAT)*		
Yes	483	84
No	91	16
*Currently*	*353*	*61*
*Not currently/ never*	*221*	*39*
History of HCV testing*		
Yes	540	95
No	27	5
*Within last 12 months*	*351*	*63*
*Not within in the last 12 months/ never*	*205*	*37*
Anti-HCV positivity (HCV RNA positive or negative)**		
Yes	432	73
No	156	27
History of HCV treatment, if anti-HCV positive*		
Yes	193	51
No	185	49
*Currently*	*23*	*6.1*
*Not currently/ never*	*355*	*94*
Viraemic HCV infection (HCV RNA positive, anti-HCV positive or negative)**		
Yes	160	27
No	428	73
HIV infected**		
Yes	14	2.4
No	568	98
HBV cleared/ infected**		
Yes	105	18
No	476	82

*self-reported, ** assessed from blood analysis, ^*§*^*analysis limited to participants with injection drug use in the last 30 days*

*Missing data in total sample: gender (1), birth region (6), years with residence in Germany (6), history of homelessness (19), current homelessness (41), history of imprisonment (11), history of imprisonment last 12 months (15), drug injection during last imprisonment (10), injections drug use last 30 days (8), main drug last 30 days (53), history of overdose (21), history of overdose last 30 days (18), years since first drug injection (17), history of shared needles/ syringes (26), history of shared needles/ syringes last 30 days (18), history of shared spoons, filters, water for drug preparation (22), history of shared spoons, filters, water for drug preparation last 30 days (14), use of sterile needle for last drug preparation (8), history of OAT (14), history of HCV testing (21), history of HCV testing last 12 months (32), history of HCV treatment (54), history of current HCV treatment (54), HIV infection (6), HBV infection (7)*

### Drug consumption experience and HCV infection risk behaviour

Median time since first IDU was 18 years (range: 0–51; n = 571), and injection of drugs in the last 30 days was indicated by 84% of participants. History of overdose with unconsciousness was disclosed by 69% of participants.

Fourteen percent of participants had shared needles/syringes during the last 30 days and 29% other equipment. The former did not differ between participants recruited in low-threshold drug services and OAT practices (12% (61/513), 95%-CI: 9.4%-15% vs. 10% (5/49), 95%-CI: 4.3%-22%). History of imprisonment was indicated by 77% of participants, and 25% of participants were imprisoned during the last 12 months. Injection drug use during last imprisonment was reported by 24% of participants. Seventy-five percent of participants reported a history of homelessness, 25% reported homelessness at the time of data collection.

### HCV infection preventive behaviour

Sixty-one percent of participants reported to be currently in OAT (type unknown). The proportion of participants who reported current OAT did not differ between participants recruited via low-threshold drug services and OAT practices (60% (316/525), 95%-CI: 56%-64% vs. 76% (37/49), 95%-CI: 62%-86%), but between participants reporting current homelessness and those who did not (42% (58/137), 95%-CI: 34%-51% vs. 69% (275/398), 95%-CI: 64%-73%). Of all participants, 93% reported to have used a new, sterile needle for the last injection.

### HCV prevalence and genotypes

Seven percent of samples were derived from venous blood and the rest from DBS. All participants who tested positive for HCV RNA also tested positive for anti-HCV. Overall, anti-HCV was detected in 73% of participants. Viraemic infection was detected in 27% (see Table 1), representing a 39% decrease from 44% in 2011–2014. Prevalence of HCV/ HIV coinfection was 1.2% (7/582, 95%-CI 0.5%-2.4%), there was no coinfection of viraemic HCV/ HBV infection observed. Proportions of viraemic and cleared infections among anti-HCV positive participants, compared to results of the DRUCK study, are shown in Fig. 1. The decrease of viraemic infections among anti-HCV positive participants was 44% (from 66% (904/1361) in 2011–2014 to 37% (160/432) in 2021–2022). Proportion of anti-HCV positive participants among all participants and proportion of viraemic infections among anti-HCV positive participants, stratified by city of recruitment, are shown in Fig. 2.

Of the 160 participants with detected viraemic infection, spots from 151 participants were available for viral sequencing. Genotyping was successful in 90 of 151 (60%). The majority of the viral sequences from the cohort were genotype 1a (n = 40; 44.4%) and 3a (n = 43; 47.8%). Other genotypes such as genotype 1b (n = 5; 5.6%), genotype 2k/1b (n = 1; 1.1%) and genotype 4d (n = 1; 1.1%) were less frequent.

Prevalence of viraemic HCV infection (among all participants) differed between male and female participants (31% vs. 19%), participants born abroad/ born in Germany (39% vs. 24%), participants reporting history of homelessness/ no history of homelessness (30% vs. 17%) and participants reporting history of imprisonment/ no history of imprisonment (31% vs. 14%). There was no difference in relation to the use of shared needles/syringes and OAT. Prevalence of anti-HCV and prevalence of viraemic infection among all participants, stratified by sociodemographic and behavioural factors, as well as HIV-/HBV-status, are shown in Table 2.

**Table 2:** Anti-HCV positivity and hepatits C viraemia, stratified by participant characteristics, DRUCK 2.0 study (2021–2022, overall sample includes 588 participants)

	Anti-HCV positivity (HCV RNA positive or negative)			Viraemic HCV infection (HCV RNA positive, anti-HCV positive or negative		
Variable	n/N	%	95%-confidence interval (CI)^#^	n/N	%	95%-confidence interval (CI)^#^
**City of recruitment**						
Augsburg	62/81	77	66.1–84.5	24/81	30	20.7–44.4
Berlin	109/143	76	68.6–82.5	40/143	28	21.2–35.9
Ingolstadt	3/4	75	23.7–96.7	1/4	25	3.3–76.3
Munich	77/105	73	64.1–80.9	16/105	15	9.5–23.5
Nuremberg	62/78	79	69.1–87.1	25/78	32	22.7–43.2
Regensburg	117/174	67	59.9–73.8	54/174	31	24.6–38.3
Wurzburg	2/3	67	15.3–95.7	0/3	0	0
**Setting of recruitment**						
Contact cafés/ outreach services	291/404	72	67.4–76.2	110/404	27	23.1–31.8
Drug consumption centres	89/112	79	71.0–86.0	35/112	31	23.4-40.4
Drug counselling centres	15/21	71	49.2–86.6	7/21	33	16.8–55.4
OAT practices	37/51	73	58.8–83.0	8/51	16	8.0–28.4
Age group*						
< 25 years	9/31	29	**15.8–47.1**	6/31	19	9.0–37.0
25–39 years	186/278	67	**61.2–72.2**	77/278	28	22.8–33.3
40 years and older	237/279	85	**80.2–88.7**	77/279	28	22.7–33.2
Gender*						
Men	293/399	73	68.9–77.5	125/399	31	**27.0–36.1**
Women	135/184	73	66.5–79.3	35/184	19	**14.0–25.4**
Diverse	4/4	100	100	0/4	0	**0**
Country of birth*						
Germany	327/458	71	67.1–75.4	109/458	24	**20.1–27.9**
Abroad	105/130	81	73.1–86.7	51/130	39	**31.2–47.9**
UN-birth region, if born abroad*						
Northern Europe	4/4	100	100	3/4	75	23.4–96.7
Western Europe	5/6	83	36.4–97.8	1/6	17	2.2–63.6
Eastern Europe	44/52	85	72.0–92.2	23/52	44	31.3–58.0
Southern Europe	14/17	82	57.0–94.3	2/17	12	2.9–37.2
outside Europe	33/45	73	58.5–84.3	17/45	38	24.8–52.7
Years with residence in Germany, if born abroad*						
Less than 5 years	7/7	100	100	4/7	57	22.7–85.8
5–9 years	18/20	90	67.3–97.5	9/20	45	25.2–66.6
10 years or more	76/97	78	69.0–85.5	34/97	35	26.2–45.1
History of homelessness*						
Yes	328/428	77	**72.4–80.4**	129/428	30	**26.0–34.7**
No	90/141	64	**55.6–71.4**	24/141	17	**11.7–24.2**
*Currently*	*110/139*	*79*	*71.6–85.1*	*60/139*	*43*	***35.2–51.5***
*Not currently/ never*	*290/408*	*71*	*66.5–75.3*	*90/408*	*22*	***18.3–26.4***
History of imprisonment*						
Yes	358/444	81	**76.7–84.1**	137/444	31	**26.7–35.2**
No	64/133	48	**39.8–56.6**	18/133	14	**8.7–20.5**
*Within last 12 months*	*122/146*	*84*	***76.6–88.7***	*65/146*	*45*	**36.7–52.7**
*Not within last 12 months/ never*	*297/427*	*70*	***65.0–73.7***	*89/427*	*21*	**17.2–25.0**
Injection drug use during last imprisonment*						
Yes	96/105	91	*67.4–93.4*	31/105	30	*17.6–49.1*
No	253/329	77	*75.3–89.2*	103/329	31	*39.2–57.5*
Injection drug use within the last 30 days*						
Yes	364/487	75	70.7–78.4	143/487	29	**25.5–33.6**
No	63/93	68	57.6–76.5	14/93	15	**9.1–23.9**
Drug used most often within the last 30 days: Opioid*						
Yes	272/376	72	67.6–76.6	110/376	29	24.9–34.1
No	122/160	76	69.0–82.2	37/160	23	17.2–30.3
Drug used most often within the last 30 days: Cocaine*						
Yes	64/76	84	74.2–90.8	19/76	25	16.5–35.9
No	329/459	72	67.4–75.6	127/459	28	23.7–32.0
Drug used most often within the last 30 days: Methamphetamine*						
Yes	33/44	75	60.2–85.6	14/44	32	19.8–46.9
No	360/491	73	69.2–77.0	132/491	27	23.1–31.0
History of overdose with unconsciousness*						
Yes	307/390	79	**74.4–82.5**	117/390	30	25.7–34.8
No	110/177	62	**54.8–69.0**	37/177	21	15.5–27.5
*Within last 30 days*^§^	*43/48*	*90*	***77.3–95.6***	*16/48*	*33*	*21.5–47.7*
*Not within last 30 days/ never*^§^	*308/421*	*73*	***68.7–77.2***	*122/421*	*29*	*24.8–33.5*
Years since first drug injection*						
Less than 2 years	11/32	34	20.1–52.1	4/32	13	4.7–29.0
2–4 years	22/42	52	37.5–66.9	14/42	33	20.8–48.7
5 years or more	385/497	77	73.6–80.9	136/497	27	23.6–31.5
History of shared needles/ syringes*						
Yes	261/320	82	**76.9–85.5**	89/320	28	23.2–33.0
No	153/242	63	**57.0–69.1**	60/242	25	19.8–30.6
*Within last 30 days *^***§***^	*50/66*	*76%*	*64.0–84.6*	*24/66*	*36*	*25.7–48.6*
*Not within last 30 days/ never *^***§***^	*300/403*	*74*	*69.9–78.5*	*111/403*	*28*	*23.4–32.1*
History of shared spoons, filters, water for drug preparation*						
Yes	316/405	78	**73.7–81.8**	117/405	29	24.7–33.5
No	98/161	61	**53.1–68.1**	34/161	21	15.5–28.1
*Within last 30 days *^***§***^	*98/138*	*71*	*62.9–78.0*	*49/138*	*36*	*28.0–43.9*
*Not within last 30 days/ never *^***§***^	*253/335*	*76*	*70.9–79.8*	*88/335*	*26*	*21.8–31.3*
Use of sterile needle for the last injection*^§^						
Yes	334/444	75	71.0–79.0	131/444	30	25.4–33.9
No	24/35	69	51.6–81.7	10/35	29	16.1–45.5
History of opioid agonist therapy (OAT)*						
Yes	373/483	77	**73.3–80.8**	130/483	27	23.1–31.1
No	48/91	53	**42.5–62.8**	23/91	25	17.4–35.2
*Currently*	*273/353*	*77*	*72.7–81.4*	*83/353*	*24*	*19.4–28.2*
*Not currently/ never*	*148/221*	*67*	*60.5–72.9*	*70/221*	*32*	*25.9–38.1*
History of Hepatitis C testing*						
Yes	408/540	76	**71.7–79.0**	149/540	28	24.0–31.5
No	13/27	48	**30.4–66.4**	7/27	26	12.9–45.3
*Within last 12 months*	*270/351*	*77*	*72.2–81.0*	*100/351*	*28*	*24.0–33.5*
*Not within in the last 12 months/ never*	*140/205*	*68*	*61.6–74.3*	*51/205*	*25*	*19.4–31.3*
HIV infected**						
Yes	12/14	86	57.2–96.4	7/14	50	25.9–74.1
No	414/568	73	69.1–76.4	151/568	27	23.0–30.4
HBV cleared/ infected**						
Yes	96/105	91	**84.3–95.5**	37/105	35	26.7–44.8
No	331/476	70	**65.2–73.5**	123/476	26	22.1–30.0

*self-reported, **assessed from blood analysis, ^§^analysis limited to participants with injection drug use during the last 30 days, ^#^Results are marked as significant (in bold) when the 95%-CIs of a variable's categories do not overlap

### HCV testing and treatment

Overall, 95% of participants reported having ever been tested for HCV. Testing during the last 12 months was reported by 63% of participants. The proportion of participants who reported testing during the last 12 months did not differ according to age, gender, history of homelessness, current homelessness, current OAT, history of imprisonment and being recruited in low-threshold drug services vs. OAT practices (results not shown). A difference was observed between participants who reported a history of previous OAT (ever been on OAT) and those who did not so (65% (302/464), 95%-CI: 61–69% vs. 50% (40/80), 95%-CI: 39%-61%), and between participants who reported being imprisoned during the last 12 months and those who did not (74% (103/139), 95%-CI: 66%-81% vs. 59% (241/408), 95%-CI: 54%-64%).

Among all anti-HCV positive participants, the proportion of cleared HCV infection and reported history of treatment was 34% (148/432). This proportion has increased since 2011–2014 (DRUCK study) from 20% (see Fig. 3). Among participants with viraemic HCV infection, reported history of treatment was 28% (45/160), indicating reinfection or unsuccessful treatment.

## Discussion

The DRUCK 2.0 study explored key indicators of HCV prevalence, risk and prevention factors, and access to care among PWID in Berlin and Bavaria for recent years. By comparing these findings with those of the DRUCK study (2011–2014), we could assess changes over the past decade, identify options for adapting public health measures and focus the future monitoring of drug-related infectious diseases among PWID in Germany.

Despite different modes of recruitment in both studies, the sociodemographic characteristics of participants in the DRUCK 2.0 study were similar to those of the participants in the DRUCK study, and also matched with findings from other studies in western European countries [4, 24, 26]. Both studies reached a high-risk population of PWID, with most participants reporting IDU in the last 30 days and other high-risk behaviours.

In the DRUCK 2.0 study, the prevalence of viraemic HCV among PWID was found to be more than 100 times higher than in the general population [36], despite an observed 44% decrease in viraemic prevalence among anti-HCV-positive individuals compared to 2011–2014. The 80% decrease in viraemic HCV prevalence, suggested as an additional indicator by WHO and EUDA [6, 7], has not yet been achieved according to our data, particularly in view of the fact that the reduction in viraemic prevalence in the overall study populations from 2011–2014 to 2021–2022 was 39%. Nonetheless, the proportion of cleared HCV infections almost doubled (34% to 63%), reflecting an increase in treated infections. This was also reported from the UK, where an increase of cleared infections from 23% to 41% among PWID was observed between 2015 and 2022 [37]. However, among those with viraemic infection, a substantial number of participants reported treatment history (28%). This may be related to treatment discontinuation or reinfection and is probably related to the high-risk population we reached. Similar findings were also observed in other studies, for example in Scotland, were rates of reinfection increased with access to treatment for those at highest risk [38].

The results of DRUCK 2.0 are, however, limited to Berlin and Bavaria and may not reflect the situation in other parts of Germany. Regional differences, such as those seen between cities in the study, could be due to heterogeneity in the composition of local study populations or of prevention, testing, and treatment services. For example, the Munich sample had a low viraemic and moderate anti-HCV prevalence compared with other Bavarian cities, suggesting low transmission due to effective harm reduction measures and/or rapid access to treatment.

Genotyping was successful in only 60% of cases, due to low viral load in a few samples and poor spot quality [32]. Notably, DBS batches that were stored at room temperature for more than a few days showed a reduced amplification efficiency (data not shown), in line with previous reports [39]. Interestingly, the data supports the observation of an increasing trend of genotype (GT) 3 infections among PWID in Germany, as previously reported [40]. This contrasts with routine GT data collected in hospitals and practices across Germany in 2016 [41], suggesting that different transmission networks exist. Furthermore, hospitalized patients may provide an insight into past transmission, whereas the current infection landscape may be quite different.

Despite the availability of new testing options since the last study, such as serological and molecular POCT, less than two-thirds of participants reported being tested within the last 12 months. The viraemic HCV prevalence did not differ by testing history, highlighting gaps in the care continuum from diagnosis to treatment initiation. Although more participants reported previous treatment compared to 2011–2014, the WHO target of 80% treatment coverage is not achieved yet, and challenges remain in ensuring access to care. Germany imposes no restrictions on HCV treatment for PWID, and costs are covered by the statutory health insurance [42]. However, only half of AIDS help- and drug services could refer people with positive antibody results to treatment providers, according to published data [43]. Potential barriers include lack of health insurance, patient non-compliance due to competing social and financial interests, as well as stigma and prejudices from medical professionals [17, 18, 43].

OAT, when used alone or in combination with NSP, is one of the most effective tools for the prevention of HCV infection and for reducing prevalence [12, 13, 21], however, in view of a shrinking heroin market and increasing cocaine and crack cocaine consumption, and availability of synthetic opioids [44], this must be put into perspective. Nevertheless, it remains one important pillar of harm reduction. The proportion of participants receiving OAT rose only slightly to reach almost two-thirds, placing Germany in the middle range among other European countries in terms of coverage [7]. New regulations allowing easier access to OAT, e.g. OAT dispensing by pharmacies and increasing take home prescription, are not yet widely implemented. With regards to NSP, the overall sharing of injecting equipment has not decreased significantly since 2011–2014, although most participants reported using sterile needles for their last injection. Limited availability of supplies contributes to this issue, as many regions in Germany do not meet the WHO targets for distribution [45].

Imprisonment is a known risk factor for HCV acquisition, [9, 46, 47] and the DRUCK study revealed associations between risk of HCV infection and length and frequency of imprisonment [48]. The current study population reported similar history of imprisonment as 10 years ago, and the viraemic HCV prevalence was found to be higher among participants reporting recent imprisonment. Access to the complete harm reduction package in prisons is limited in Germany [49, 50], with only one prison in Berlin offering NSP, and with inconsistent implementation of OAT, HCV testing and treatment.

Worryingly, homelessness, a growing public health concern in Germany [51], has doubled among study participants since 2011–2014. A study conducted in 2021 among people experiencing homelessness in Berlin revealed a high overlap between homelessness and IDU, as well as high HCV prevalence, low treatment coverage, and significant barriers to accessing healthcare (e.g. lack of health insurance), including OAT [52]. Associations between homelessness and HCV among PWID have been described previously [53], and from DRUCK 2.0 we confirm a higher viraemic HCV prevalence and lower OAT coverage among participants reporting current homelessness in comparison to others.

The study acknowledges some limitations: data was collected in the facilities by convenience sampling, which may have led to non-representative results. Participants without German language skills were underrepresented and the language skills of staff might have influenced the composition of the migrant population reached by each facility. In contrast to DRUCK 2.0, participants of the DRUCK study (2011–2014) were recruited through RDS, which may have reached more PWID outside the state- and civil-society led support systems. Moreover, the sample of the DRUCK study was quite larger than the DRUCK 2.0 sample and drawn from more federal states, leading to more valid data. Despite this larger sample, the results from 2011–2014 were published without the respective CIs, but instead with city ranges, to account for heterogeneity across the study cities [54]. However, this limits a more detailed comparison with the results of DRUCK 2.0 for 2021–2022 and only allows a comparison of the point estimate without using CIs. DBS testing, although convenient, was slightly less sensitive than venous blood testing, with a sensitivity of 97% for anti HCV (specificity: 100%). For RNA analysis the limit of detection for DBS with approximately 1000 IU/ml is also reduced compared to venous blood samples (4 IU/ml), mainly restricted by the quality of DBS [55]. However, all participants were tested for both anti-HCV and HCV RNA to limit the constraints. The proportion of those diagnosed and receiving treatment could not be calculated, as the timing between diagnosis and treatment could not be derived from the data. This may lead to an underestimation of treatment coverage in relation to the WHO target regarding treatment coverage. Other limitations include potential bias in self-reported data, issues with answering sensitive questions, and challenges related to the COVID-19 pandemic affecting service availability [56].

Despite significant progress in eliminating HCV among PWID, major gaps remain, particularly in access to prevention, early diagnosis and treatment.

Harm reduction services in Germany need to be significantly strengthened. Municipalities and federal states should increase NSP funding to promote safer use. Access to OAT should be further facilitated, with a focus on availability and accessibility according to WHO and UNODC standards [57]. In particular, low-threshold OAT distribution and the provision of naloxone kits to prevent drug-related deaths as part of a harm reduction package should be rolled out nationwide [20, 58].

Low-threshold HCV testing services for PWID should be further expanded, with a focus on options for on-site HCV PCR testing given the high proportion of PWID with a history of infection. Information and counselling at the time of diagnosis is essential to promote treatment. Particular attention should be paid to the possibility of reinfection. Promotion of regular testing, also after cure of an HCV infection, and reducing the stigma of multiple treatment courses are important to address this issue, particularly in high risk and most vulnerable subgroups [58, 59].

Promoting HCV treatment uptake among PWID requires a multi-level approach, including local networks between low-threshold drug services and infectious disease specialists, as well as social workers or peers to guide infected people through the treatment process [60]. Good local practice already exists in Germany [61] and should be scaled up as shown in other countries. In addition, with the new HCV treatment regimens, it is now reasonable to move HCV treatment into general practice and even into community settings, such as drug services and pharmacies [62–64]. Low-threshold “one-stop-shop” services that integrate HCV screening and treatment, as well as non-invasive assessment of liver damage may help to further improve treatment and prevent both late sequelae and transmission. Successful models are reported from other countries, e.g. Norway and Scotland, including on-site testing and treatment offered in mobile HCV clinics, as well as scale-up in other community drug services for micro-elimination [65, 66].

Overall, opportunities to increase access to HCV prevention and care for PWID should be fully exploited. Existing points of contact between PWID and the health system, such as OAT, have to be better used for HCV testing and treatment [67]. In prisons, availability of harm reduction services, regular informed hepatitis testing, and consecutive treatment initiation should be standard [68]. We found a higher proportion of recent HCV testing in those recently imprisoned in comparison to those who were not, hinting at the opt-out testing policy in Bavarian prisons. If followed by a treatment offer in those who tested positive, this can be an opportunity and should be expanded. An HCV treatment model is currently being piloted in three German prisons and urgently needs to be expanded to all prisons [49]. Strong evidence from other countries is available and should be considered in Germany, e.g. the PIVOT study from Australia, showing that a “one-stop-shop” prison intervention (point-of-care HCV testing and a nurse-led evaluation before treatment initiation) can enhance treatment uptake and reduce time to treatment initiation among people recently imprisoned [69]. Telemedicine as a novel approach can further increase treatment uptake in prison settings, but also in remote regions [70].

 Finally, when implementing prevention and care services for PWID, particular attention must be paid to severely underserved subpopulations. For example, better networking between drug and homelessness services and targeted approaches, such as the provision of OAT and HCV treatment in shelters, can improve access to HCV prevention and care for homeless PWID [71]. Further, most importantly, universal health coverage with unbureaucratic coverage of treatment costs, including for people without health insurance, is the critical foundation for access to HCV care.

In conclusion, achieving HCV elimination in Germany until 2030 requires more efforts and adequate funding to expand innovative models of care and scale up prevention, testing and treatment provision for PWID. The targets of an 80% reduction in viraemic prevalence among PWID and 80% treatment coverage have not yet been reached. Achieving these targets requires universal health coverage and targeted integrated testing and treatment for those most at risk, such as PWID and people experiencing homelessness. PWID receiving OAT and people in prisons should be offered testing and treatment at any contact with the medical system. The nationwide monitoring system for drug-related infectious diseases, based on the DRUCK 2.0 study design, is currently being implemented. The new round of data collection in 2025 will help to re-assess the coverage of and access to interventions and track progress towards elimination of HBV, HCV and HIV among PWID in Germany [30].

## Supplementary Information

Additional file. 1

## Data Availability

The datasets used and/or analysed during the current study are available from the corresponding author on reasonable request.

## Abbreviations

EUDA: European Union Drugs Agency
HBV: Hepatitis B virus
HCV: Hepatitis C virus
HIV: Human immunodeficiency virus
IDU: Injection drug use
NSP: Needle syringe program
OAT: Opioid agonist treatment
POCT: Point of care testing
PWID: People who inject drugs
RDS: Respondent driven sampling
RKI: Robert Koch Institute
WHO: World Health Organization
