# Supplementary material for: Progress and challenges in the elimination of hepatitis C among people who inject drugs in Germany: results of a pilot study for a national monitoring system, 10 years after the first data collection
Source: Harm Reduct J. 2024 Dec 20;21:222. doi: 10.1186/s12954-024-01119-2 (PMC11660851; doi:10.1186/s12954-024-01119-2)
Supplement: Supplementary file 1 — Additional file. 1 [file 12954_2024_1119_MOESM1_ESM.docx]

**Additional file 1:** Flowchart of the study population, DRUCK 2.0 study (2021-2022)

**72 participants did not fulfil inclusion criteria 35** RKI didn´t receive questionnaire/ consent form **2** laboratory didn't receive a blood sample **22** denied drug injection in the last 12 months **13** missing information on drug injection in the last 12 months/ age

**6** participants with missing anti-HCV and/or HCV-RNA status

**588 participants with determined infection status**

**596 participants fulfilled inclusion criteria**

**668 participants**
